# A Quality Improvement Initiative to Reduce Pediatrician Burnout Led by the American Academy of Pediatrics Section on Internal Medicine and Pediatrics (Med-Peds)

**DOI:** 10.7759/cureus.41205

**Published:** 2023-06-30

**Authors:** Tina Y Hu, Abhishek Surampudy, Himani Divatia, Allen R Friedland

**Affiliations:** 1 Internal Medicine-Pediatrics, ChristianaCare/Nemours Children's Hospital, Delaware, Newark, USA; 2 Internal Medicine-Pediatrics, ChristianaCare, Newark, USA

**Keywords:** med-peds, physician burnout, quality improvement, american academy of pediatrics, gender, physician wellness, preventive health, internal medicine-pediatrics

## Abstract

Physician burnout impacts care (of self and patient), productivity, longevity of career, and overall cost to the system. While burnout rates for pediatricians are lower than average, they have not improved significantly over time. While strategies at the system level have been more successful than those at the individual level, both aspects are vital.

This quality improvement study explores physician wellness and burnout trends of a sample population of pediatricians at the 2018 and 2019 AAP National Conference and Exhibition (NCE), using the Physician Health and Wellness Booth (PHWB). A rapid cycle approach with the Plan-Do-Check-Act (PDCA) framework was utilized. The aim was to observe if reported burnout decreased by 20% over six months.

Of the pediatricians who interacted with the PHWB, 56 were randomly selected to participate. This included men and women and those in various practice settings, ranging from resident physicians to providers in practice for over 20 years. Baseline surveys included elements from a modified Maslach Burnout Inventory and the Stanford Physician Wellness Survey, focusing on burnout components (emotional exhaustion, depersonalization, and fulfillment) and wellness activities. Individual-based interventions were provided at the PHWB, including adult preventative health guidelines, resources on sleep, stress mitigation, and complementary medicine. Participants received a movie ticket and Starbucks gift card. Follow-up included six monthly newsletters with strategies from seven wellness domains. Post-intervention surveys at six months assessed all baseline questions plus the effectiveness of monthly newsletters.

A second PDCA cycle was conducted from the 2019 NCE. All individual-based interventions continued with an added aromatherapy oil station. Additional system-based resources included sample institutional wellness initiatives and burnout cost analyses, all focusing on advocating for cultural change at their respective home organizations. Interactive monthly wellness calendars addressing seven wellness domains were emailed for six months follow-up.

Results from 10 post-intervention surveys (10/56=18% of respondents) from the initial cohort reported an average of 25% decrease in burnout (p=0.09). This was measured on a scale of 1-10 (from “never” burned out to “very often”) and improved from 6.68 (“sometimes” to “often” burned out) to 5.0 (“rarely” to “sometimes” burned out). Results from Cohort 2 reflected a decrease in burnout from 4.94 (“rarely” to “sometimes” burned out) to 2.85 (“never” to “rarely” burned out) in return from 20 post-intervention surveys (20/48=42% of respondents, p=0.003). Participants noted a lack of control over work schedules and a disconnect with organizational values as drivers of burnout. Both the PHWB and monthly newsletters were rated as valuable as reminders about wellness practices.

Limitations included low response rate, which was notable, and inability to prove causation of improvement from our intervention. Future steps include utilizing subject identification numbers to allow for anonymity in a prospective cohort study with a third PDCA cycle. This would allow anonymous but matched same-subject comparison of pre- and post-survey results despite the small sample size. Follow-up incentives could be beneficial. Lastly, data from both cohorts revealed the highest level of burnout in early career physicians within 10 years of training, paving an opportunity for future study.

## Introduction

Physician burnout impacts care (of self and patient), productivity, career longevity, and overall cost to the healthcare system with significant levels of burnout that continue to persist over the years [1-4]. While burnout rates for pediatricians are lower than the average for physicians in other specialties (41%), the rates have not improved significantly over time [5]. Many strategies have been studied to combat physician burnout, with initial efforts targeted at the individual level and improving resilience. Individual-based strategies include optimizing personal factors, skills and abilities, and healthcare roles and responsibilities [6]. However, emerging research has shown the value of interventions targeted at the organizational level. Many studies suggest that interventions at the organizational level are more successful than those at the individual level in reducing burnout, although both aspects are vital [6-12]. System-based strategies include optimizing practice efficiency, organizational culture, community and teamwork, leadership, control and autonomy, and work-life integration, among others [6,10-14].

Female physicians face unique challenges that may contribute to burnout [15]. Although recent studies found that female physicians reported a higher prevalence of burnout than male physicians in both academic practice (50.7% vs 38.2%, p<0.0001) and private practice (48.1% vs 40.7%, p=0.001) settings, the multivariable analysis found no statistically significant gender-based differences [16]. There are limited data specific to pediatricians and also to gender differences among pediatricians [2,17-21]. 

In 2010, the AAP Section on the Internal Medicine and Pediatrics (Med-Peds) Physician Health and Wellness (PHW) group was created to be part of the yearly exhibit hall of the AAP National Conference and Exhibition (NCE) to provide basic information about preventive health guidelines to pediatrician attendees who may not be focused or up-to-date on their own healthcare [22].

In 2018, a quality improvement project (QI) was added to focus on wellness and burnout in a sample population of pediatricians attending the 2018 or 2019 AAP-NCE, utilizing the newly created J. H. Milligan-Barr Physician Health and Wellness Booth (PHWB) [22].

The aim of the QI project was to see if reported burnout decreased by 20% over a six-month period in both cohorts through individual and organizational-based strategies at the PHWB and to report on any gender differences.

## Materials and methods

This study was reviewed and approved by the AAP Institutional Review Board (IRB), Protocol number 19 DI 01.

Cohort 1

In October 2018, the PHW group identified the first cohort of pediatricians attending the conference and visiting the PHWB. Over 200 attendees visited the PHWB, and 56 participants were randomly selected to be followed for six months. All PHWB visitors were asked about their desire to participate, and randomization was strictly based on including those who demonstrated self-generated interest to participate in the QI initiative, completed the pre-survey, and agreed to monthly follow-up. 

Intervention 1

A pre-survey was completed at the PHWB just prior to the on-site intervention, and a follow-up survey was completed at the end of the six-month period. The survey was compiled from a variety of sources including the Maslach Burnout Inventory and Stanford Physician Wellness Survey [21-23]. Pre-survey questions comprised topics related to self-assessment of burnout, emotional exhaustion, depersonalization, wellness activities, and workplace autonomy and contributors to burnout. The post-survey included these questions and additional questions related to the ongoing impact of the PHWB and wellness strategies.

The materials for interventions included initiatives on-site at the AAP NCE, as well as those delivered electronically during the follow-up period of six months. Interventions focused on individual-based strategies. On-site initiatives included education about adult preventive healthcare guidelines, stress reduction techniques, strategies to enhance sleep, and information on integrative medicine. Each participant also received a $25 Starbucks gift card and a movie ticket for their participation. At monthly follow-up, each participant received a health and wellness electronic newsletter highlighting strategies to promote wellness in all seven domains focusing on individual-based strategies, including physical wellness, emotional wellness, spiritual wellness, social wellness, environmental wellness, financial wellness, and intellectual wellness (see Appendices). The participants were encouraged to incorporate these individual strategies into their weekly wellness routines.

Cohort 2

In October 2019, the PHW group identified the second cohort of pediatricians attending the conference and visiting the PHWB. Over 250 attendees passed by the PHWB, and 48 participants were randomly selected to be followed for six months. All PHWB visitors were asked about their desire to participate, and randomization was strictly based on including those who demonstrated self-generated interest to participate in the QI initiative, completed the pre-survey, and agreed to monthly follow-up. 

Intervention 2

The second cohort completed similar pre- and post-surveys at six months following the initial intervention. Due to findings of poor organizational support as a contributor to burnout from the first Plan-Do-Check-Act (PDCA) cycle in Cohort 1, additional interventions were added to address systemic factors and resources for physicians to advocate for wellness. In addition to education on preventive healthcare guidelines, stress reduction techniques, and strategies to enhance sleep, each participant was invited to sample essential oils and learn about the beneficial effects of aromatherapy. Furthermore, they received specific resource guides and cost analyses of wellness initiatives to advocate for within their respective institutions to advocate for and promote organizational change in establishing a culture of wellness. Each participant also received a sample of essential oils in addition to a $25 Starbucks gift card. Monthly follow-up over a six-month period continued in a similar manner as that of Cohort 1, except for the development of a wellness calendar in place of a wellness newsletter (see Appendices).

The pre- and post-surveys were analyzed, and the rates of burnout and impact of interventions were compared between both cohorts using t-tests to calculate the statistical significance. Additional trends in demographics, including gender and burnout rates, were also analyzed. 

## Results

The pre-survey completion rate for Cohort 1 was 100% (56/56), and the post-survey completion rate for Cohort 1 was 18% (10/56). The pre-survey completion rate for Cohort 2 was 100% (48/48), and the post-survey completion rate for Cohort 2 was 42% (20/48). 

Demographics

This prospective cohort study evaluated 56 pediatricians at the 2018 and 48 pediatricians at the 2019 NCE. Both cohorts consisted of a majority of female participants (66% in Cohort 1 and 90% in Cohort 2). In both cohorts, the highest proportion of participants was those in a practice associated with a medical school or parent university (21% and 35% in Cohorts 1 and 2, respectively), followed closely by those in a nongovernment hospital or clinic and a multi-specialty group practice. No participants worked for government hospitals or clinics across both cohorts. In regard to participants and years of experience, the highest proportion of participants was those with fewer than 5 years of experience (34% in Cohort 1 and 40% in Cohort 2), while those with over 20 years of experience comprised 29% of the population in each of the cohorts. Table 1 highlights the full demographic details of the participants in both cohorts.

**Table 1:** Demographics

	Cohort 1 N=56	Cohort 2 N=48	Mean	Standard Deviation
Female	66%	90%	78%	0.12
Male	34%	10%	22%	0.12
Practice Location:				
Medical school (or parent university)	21%	35%	28%	0.07
Multispecialty group practice (other than staff model HMO)	12%	17%	15%	0.025
Nongovernment hospital/clinic	13%	10%	12%	0.015
Nonprofit community health center	9%	0%	5%	0.045
Solo or two-physician practice	2%	10%	6%	0.04
Pediatric group practice of 3-5 pediatricians	9%	4%	7%	0.025
Pediatric group practice of 6-10 pediatricians	8%	6%	7%	0.01
Pediatric group practice of >10 pediatricians	9%	8%	9%	0.005
US government hospital/clinic	0%	0%	0%	0
City/county/state government hospital/clinic	0%	7%	4%	0.035
Not specified	12%	0%	6%	0.06
Other patient care or non-patient care employment	5%	3%	4%	0.01
Years of Experience (after completion of training):				
< 5 years	34%	40%	37%	
5 to 10 years	9%	8%	9%	
10 to 15 years	11%	10%	11%	
15 to 20 years	17%	13%	15%	
> 20 years	29%	29%	29%	

Overall burnout levels

**Figure 1:**
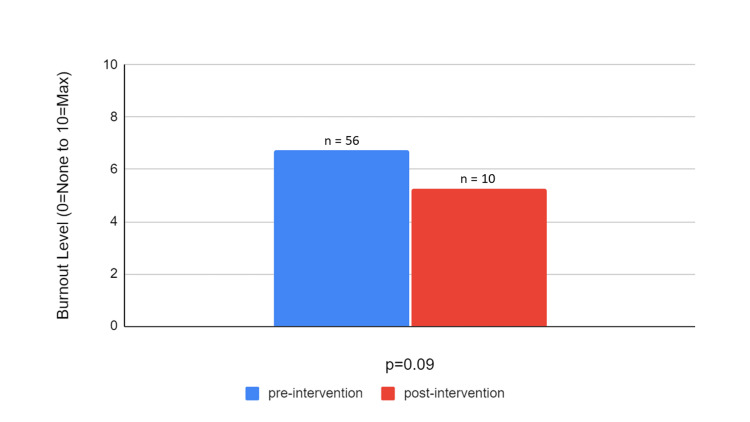
Cohort 1 Pre- and Post-Intervention Burnout Levels

**Figure 2:**
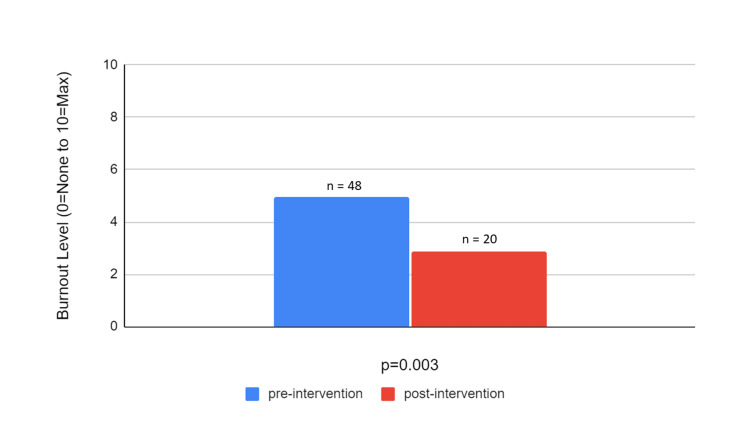
Cohort 2 Pre- and Post-Intervention Burnout Levels

As demonstrated in Figure 1, the initial burnout levels in Cohort 1 were 6.74, which decreased to 5.25 (on a scale of 0-10 from no burnout to the maximum level of burnout) following individual-based wellness interventions. While rates of burnout decreased, results were not statistically significant (p=0.09). Figure 2 presents the pre- and post-intervention burnout levels in Cohort 2. In Cohort 2, the initial burnout levels were 4.94, which decreased to 2.85 following combined individual- and organizational-based wellness interventions that were statistically significant (p=0.003).

Burnout levels by gender

**Figure 3:**
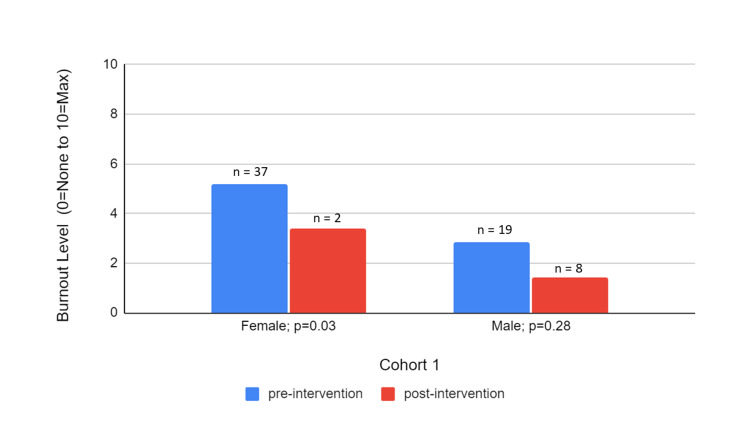
Cohort 1 Pre- and Post-Intervention Burnout Levels by Gender

**Figure 4:**
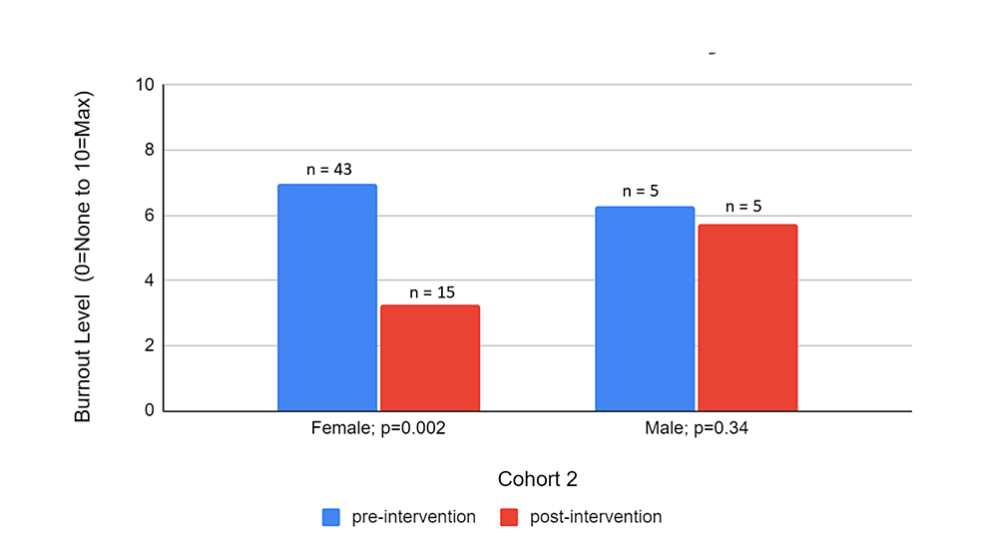
Cohort 2 Pre- and Post-Intervention Burnout Levels by Gender

The mean burnout levels decreased for both genders' post-intervention in both Cohorts 1 and 2 as depicted in Figures 3-4, respectively. It is notable, however, that the decrease is statistically significant only in women across both cohorts (from 5.18 to 3.42 in Cohort 1 and from 6.97 to 3.25 in Cohort 2) although the sample size of the female respondents on the post-survey of Cohort 1 was only two. Overall, the mean burnout levels were also higher in Cohort 2 than in Cohort 1 initially and after wellness interventions. In Cohort 2, there was a similar percentage decrease in burnout across both genders and a statistically significant larger improvement in burnout for women compared to men.

Monthly wellness follow-up

The qualitative data on the monthly wellness follow-up for Cohorts 1 and 2 demonstrated an overall positive attitude toward monthly follow-up and reminders for wellness, but responses were very limited, and there was no significant change in individual practice habits due to the monthly follow-up. The Appendices include samples of the monthly wellness newsletter and the monthly wellness calendar.

## Discussion

In this prospective cohort study, with each of the two cohorts examining 56 and 48 participants over nine months for Cohorts 1 and 2, respectively, we examined the overall burnout level and additional variables in wellness.

Overall, the majority of participants were female, with Cohort 1 more closely resembling the distribution of females and males in pediatrics than in Cohort 2. In regard to the practice setting, the sample of pediatricians had a higher proportion of those in a practice associated with a medical school or parent university, which was slightly less than the national average [23].^ ^The distribution of pediatricians at various times in their careers reflects that of the general population [23].

The mean burnout levels improved in both cohorts, suggesting a level of success from the intervention (a decrease of 22% in Cohort 1 and 42% in Cohort 2), although the improvement was statistically significant only in Cohort 2. This suggests that the intervention in Cohort 2, which included a combined institutional- and individual-based intervention, was associated with a more effective reduction in burnout than a sole individual-based intervention. It is important to note that the study period for Cohort 2 ran into the start of the COVID-19 pandemic with the exact impact on our study results unclear; it is possible that the pandemic was associated with higher rates of burnout during the middle and end of the study, in which case our combined wellness intervention may have been even more effective with lower post-intervention burnout rates than without the effects of the pandemic. It is possible as there is increasingly more focus on physician wellness and burnout, and resources from institutional, external, or even national influences may have led to improvement in burnout over the years.

There was also overall higher average burnout in women than in men both before and after the intervention, which is consistent across both cohorts. This may suggest that women are at higher risk for burnout than men. Still, for both genders, there was a mean decrease in burnout; however, the improvement was more marked and statistically significant in females in Cohort 2 (53% improvement with p=0.002). This may suggest that women are more prone to benefit from our specific interventions or the overall type of interventions. It seems that women benefit from individual-based interventions, which were present for both cohorts, but benefit to a greater extent with system advocacy resources included.

Limitations included a poor follow-up rate of 10 (18%) and 20 (43%) participants in Cohorts 1 and 2, respectively, which is just at or below the national average for the response rate of prospective QI surveys. Additionally, there may have been a component of selection bias with many participants lost to follow-up and those who fared better in wellness choosing to respond to the post-intervention survey. The second cohort included a follow-up time that was amid the COVID-19 pandemic with a possible decreased response rate. Affinity bias could have also occurred with participants selectively approaching the vicinity of the PHWB in order for staff to randomly select them. These participants may have preexisting interests in wellness, as opposed to those who did not approach or chose not to participate. In the follow-up survey, we did not ask if the participants incorporated the individual wellness interventions into their lifestyle or used organizational-based resources to advocate for physician wellness at their organizations. Therefore, it is difficult to conclude the exact factors that contributed to their improvement in burnout. Finally, there is a possible sampling bias with fewer males included in the study in Cohort 2 when considering the gender of all pediatricians in the United States; however, given the sample population at the AAP NCE, which included more females, the sampling at the conference itself may have not been as disproportionate. 

Future directions include a prospective study with random selection of participants at a larger forum to eliminate the limitation of a small physical venue for recruitment; expanding studies and initiatives in early career physicians (fewer than five years after training); collaborating with other AAP sections or committees; and extending interventions across various practice settings. As there may have been confounding factors, future studies are also needed. These include examining the impact of the sole presence of the PHWB on a national platform, which may demonstrate system support for physician wellness; intrinsic motivation for wellness inspired by and distinct from PHW initiatives; and PHW system-based resources. 

## Conclusions

Physician burnout is increasingly prevalent among healthcare providers, and pediatricians are not immune to this phenomenon. While the rates of pediatrician burnout have not increased significantly on a national platform, the paucity of increasing wellness and persistent feelings of emotional exhaustion and depersonalization inhibit optimal performance and personal and professional satisfaction. Furthermore, data suggest that organizational factors contribute to physician burnout at a higher level than individual factors. Developing a QI initiative organized by the AAP Section on Med-Peds provided an opportunity for a select number of pediatricians attending the AAP NCE to receive individualized wellness education and preventive health strategies while also providing resources to advocate for organizational wellness systems and culture. Med-Peds physicians served the unique role of being "the internist for the pediatrician" and empowered individuals within each cohort of this initiative to enhance the focus on their own wellness and achieved the aim of reducing burnout by 20% in a six-month period. 

## Figures and Tables

**Figure 5:**
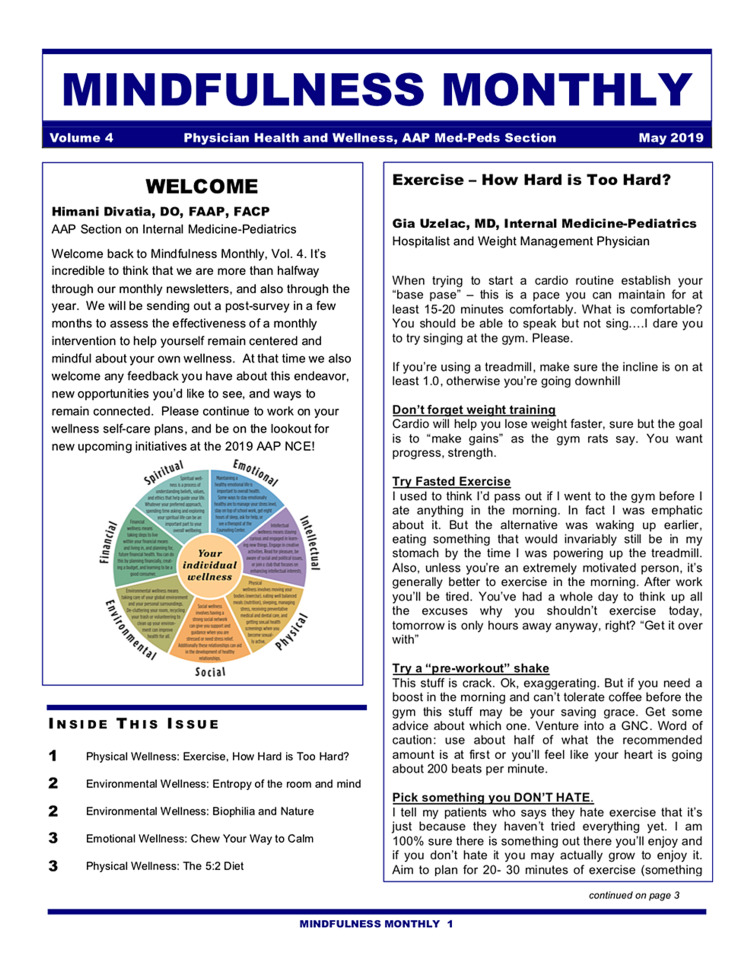
"PHW Mindfulness Monthly" Monthly Newsletter

**Figure 6:**
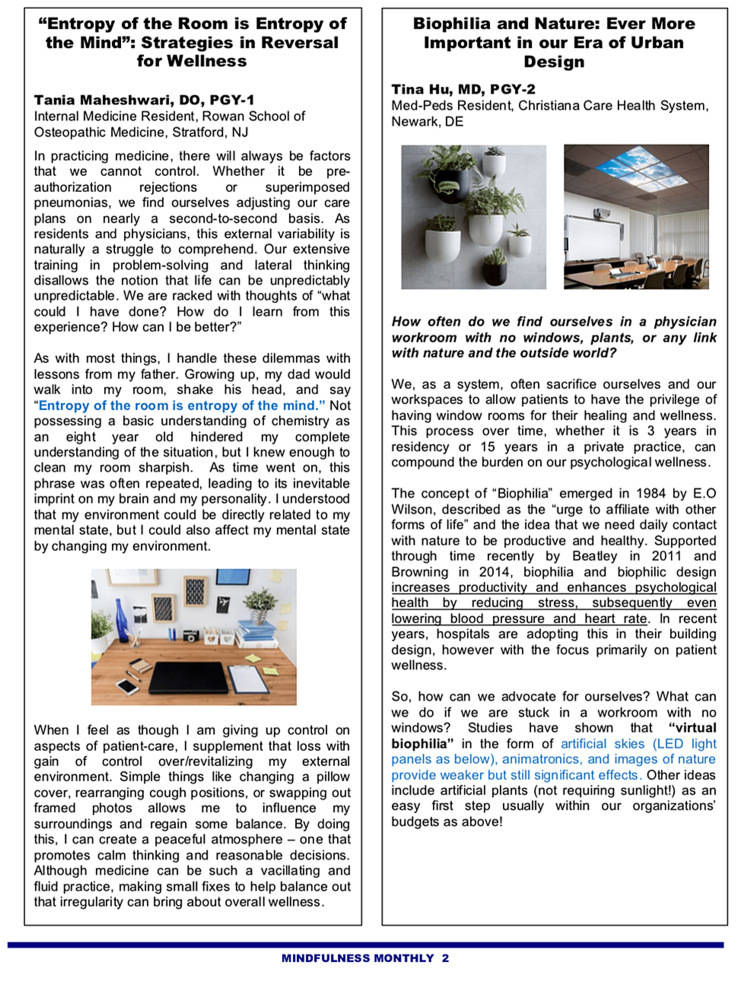
PHW "Mindfulness Monthly" Monthly Newsletter

**Figure 7:**
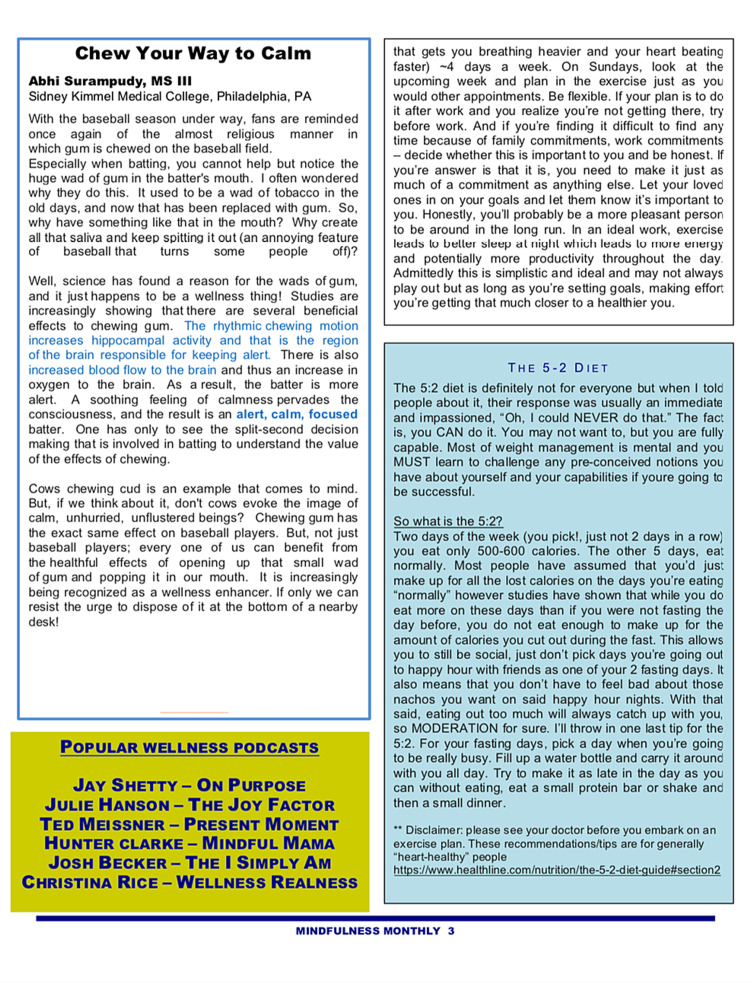
PHW "Mindfulness Monthly" Newsletter

## References

[1] Shanafelt TD, Boone S, Tan L. (2012). Burnout and satisfaction with work-life balance among US physicians relative to the general US population. Arch Intern Med.

[2] Shanafelt TD, Hasan O, Dyrbye LN, Sinsky C, Satele D, Sloan J, West CP (2015). Changes in burnout and satisfaction with work-life balance in physicians and the general US working population between 2011 and 2014. Mayo Clin Proc.

[3] Han S, Shanafelt TD, Sinsky CA (2019). Estimating the attributable cost of physician burnout in the United States. Ann Intern Med.

[4] West CP, Dyrbye LN, Shanafelt TD (2018). Physician burnout: Contributors, consequences and solutions. J Intern Med.

[5] Kane L. 2019 (2020). Medscape national physician burnout, depression, and suicide report 2019. https://www.acam.org/news/444019/Medscape-National-Physician-Burnout-Depression--Suicide-Report-2019.htm.

[6] Olson K, Marchalik D, Farley H (2019). Organizational strategies to reduce physician burnout and improve professional fulfillment. Curr Probl Pediatr Adolesc Health Care.

[7] De Simone S, Vargas M, Servillo G (2021). Organizational strategies to reduce physician burnout: A systematic review and meta-analysis. Aging Clin Exp Res.

[8] Shanafelt TD, Gorringe G, Menaker R (2015). Impact of organizational leadership on physician burnout and satisfaction. Mayo Clin Proc.

[9] Shanafelt TD, Noseworthy JH (2017). Executive leadership and physician well-being: Nine organizational strategies to promote engagement and reduce burnout. Mayo Clin Proc.

[10] Shanafelt TD, Dyrbye LN, West CP (2017). Addressing physician burnout: The way forward. JAMA.

[11] Rothenberger DA (2017). Physician burnout and well-being: A systematic review and framework for action. Dis Colon Rectum.

[12] Deckard G, Meterko M, Field D (1994). Physician burnout: An examination of personal, professional, and organizational relationships. Med Care.

[13] Brigham TC, Barden AL, Dopp A (2023). Brigham TC, Barden AL, Dopp A, et al. https://nam.edu/journey-construct-encompassing-conceptual-model-factors-affecting-clinician-well-resilience/.

[14] Tawfik DS, Profit J, Webber S, Shanafelt TD (2019). Organizational factors affecting physician well-being. Curr Treat Options Pediatr.

[15] Marshall AL, Dyrbye LN, Shanafelt TD (2020). Disparities in burnout and satisfaction with work-life integration in U.S. physicians by gender and practice setting. Acad Med.

[16] McMurray JE, Linzer M, Konrad TR, Douglas J, Shugerman R, Nelson K. (2000). The work lives of women physicians results from the physician work life study. The SGIM Career Satisfaction Study Group. J Gen Intern Med.

[17] Rabatin J, Williams E, Baier Manwell L, Schwartz MD, Brown RL, Linzer M (2016). Predictors and outcomes of burnout in primary care physicians. J Prim Care Community Health.

[18] Jager AJ, Tutty MA, Kao AC (2017). Association between physician burnout and identification with medicine as a calling. Mayo Clin Proc.

[19] Maslach C, Jackson SE, Leiter MP (1997). The Maslach burnout inventory manual - Third edition. Evaluating Stress: A Book of Resources.

[20] Trockel M, Bohman B, Lesure E, Hamidi MS, Welle D, Roberts L, Shanafelt T (2018). A brief instrument to assess both burnout and professional fulfillment in physicians: Reliability and validity, including correlation with self-reported medical errors, in a sample of resident and practicing physicians. Acad Psychiatry.

[21] (2023). Stanford physician wellness survey. https://documents.christianacare.org/Stanford-Physician-Wellness-%20Measures_9-2-2016.pdf.

[22] Hu TY, Surampudy A, Friedland AR, Divatia H (2021). Balancing burnout: Assessing and improving physician health and wellness. Pediatrics.

[23] (2023). American Academy of Pediatrics, Periodic Survey, 2018-2019. Pediatricians’ practice and personal characteristics: Pediatricians’ employment setting distribution. https://downloads.aap.org/AAP/Images/setting.png.

